# South Asian Society for Sexual Medicine School, Bangladesh: a multidisciplinary training initiative in an under-resourced country

**DOI:** 10.1093/sexmed/qfaf028

**Published:** 2025-04-30

**Authors:** Mohammad Shamsul Ahsan, Leuza Mubassara, A K M Anwarul Islam, Md Mahbubul Hasan, Shahjada Selim, Rubaiya Ali, Mohtasham Hasan, Md Kamrul Hossain

**Affiliations:** Department of Psychiatry, Bangabandhu Sheikh Mujib Medical University, Dhaka 1000, Bangladesh; Department of Psychiatry Dhaka Bangladesh, Bangabandhu Sheikh Mujib Medical University, Dhaka 1000, Bangladesh; Department of Urology, Bangabandhu Sheikh Mujib Medical University, Dhaka 1000, Bangladesh; Department of Psychiatry, Jashore Medical College Hospital, Jashore, Bangladesh; Department of Endocrinology, Bangabandhu Sheikh Mujib Medical University, Dhaka 1000, Bangladesh; Department of Dermatology and Venerology, Evercare Hospital, Dhaka 1000, Bangladesh; Department of Child, Adolescent and Family Psychiatry, National Institute of Mental Health (NIMH), Dhaka 1000, Bangladesh; Department of Computer Science and Engineering, Daffodil International University, Dhaka 1000, Bangladesh

**Keywords:** training program, knowledge, SASSM, sexual medicine, Bangladesh

## Abstract

**Background:**

The yearly courses conducted by the South Asian Society for Sexual Medicine (SASSM) in Bangladesh establish a collaborative training paradigm designed to increase healthcare professionals’ comfort with sexual medicine and develop skills for giving the best treatment possible to individuals with sexual health issues and dysfunctions.

**Aim:**

The study assessed the impact of course completion on SASSM School Bangladesh participants.

**Methods:**

A pretest–posttest of 116 (57%) SASSM participants was conducted, comprising data on knowledge acquisition following the 3-day program in 2017, 2018, 2019, and 2021. A paired *t*-test was applied to compare the difference between pretest and posttest performance. Logistic regression was conducted to assess the influence of socio-demographic variables.

**Outcomes:**

The study demonstrates that the SASSM School Bangladesh program significantly improves participants’ knowledge of sexual medicine, with a 12.7-unit increase in test scores, irrespective of socio-demographic factors.

**Results:**

Majority (102, or 75% of those polled) of the participants were men. Psychiatrists were the most common respondents (39%), followed by dermatologists (32%), urologists (12%), and gynecologists (8%) who completed both surveys. There was a significant difference (*P*-value < .01) in mean pretest (49.6 ± 17.1) and posttest (62.3 ± 15.5) scores. The socio-demographic factors (gender, location, age, and experience) do not have any significant influence on gain of knowledge through the SASSM training program as the *P*-value of odds ratio or adjusted odds ratio from logistic regression is *P* > .05.

**Clinical Implications:**

The study highlights the clinical importance of structured training in sexual medicine, ensuring that healthcare professionals across various specialties are better equipped to diagnose and manage sexual health issues effectively.

**Strengths and Limitations:**

The study used a pretest–posttest design to measure actual knowledge improvement. Paired *t*-test and logistic regression were applied to ensure robust analysis. Diverse professionals from psychiatry, dermatology, urology, and gynecology participated in the study, making the findings relevant across specialties. This study evaluates the only structured sexual medicine training program in Bangladesh, contributing new insights into medical education.

However, the findings may not be generalizable beyond Bangladesh. The study could not assess whether knowledge retention and clinical practice improved over time. Participants voluntarily enrolled in the study, which can possibly represent a group already interested in sexual medicine. The study focused only on knowledge improvement, not on changes in clinical practice or patient outcomes.

**Conclusion:**

Specialists of sexual medicine have a unique opportunity to learn and certify their knowledge through the SASSM program, the first of its kind in Bangladesh.

## Introduction

Sexual dysfunctions are global phenomena affecting 43% of men and 49% of women with at least one problem,^1^ whereas in Asia, over 20% of men and 30% of women reported sexual problems.^2^ In addition to reducing quality of life, sexual dysfunctions increase the risk of divorce, mild depression, generalized anxiety disorder, mixed anxiety depression, and nicotine dependence.^3^ Studies show that among those who need care in this regard, very few people actually get it. Due to cultural stigma, ignorance, miscommunication, and a fear of upsetting patients, doctors frequently hesitate to have open discussions with their patients about sexuality.^4^ A study conducted among a sample of physicians in Bangladesh indicated that 53.6% were comfortable and 35% were uncomfortable asking about sexual history, with 26% experiencing discomfort, 12% apprehensive with extreme age, and 12% fearful of offending patients.^5^ South Asia falls short of independent medical specialties such as sexology or sexual medicine, offering formal education on sexual health.^6^ The field of sexual medicine is run by specialists of various fields including urologists, dermatologists, endocrinologists, general practitioners, obstetricians, and psychiatrists.

Considering the European School of Sexual Medicine as a center of excellence,^7^ the SASSM was founded with a multidisciplinary team. The SASSM Bangladesh was established with a very important contribution from the ISSM Zorgniotti grant on two different occasions in 2017 and 2019. Under the direction of the South Asian School of Sexual Medicine (SASSM) board, members of SASSM in Bangladesh launched a 3-day modern teaching program first in September 2017 and eventually in 2018 and 2019.^8^ Faculty members from South Asia, Turkey, and various European countries, including the United Kingdom, used to be present.

A total of 203 Bangladeshi physicians from various fields have received certificates from the annual course so far. The purpose of this study is to assess the impact of course completion on SASSM School Bangladesh participants. Thus the alternative hypotheses for the study were (1) H_A1_: There is significant knowledge gain through the SASSM training program and (2) H_A2_: The socio-demographic variables (gender, location, age, and experience) have a significant impact on knowledge gain after the program. This study will allow for a better understanding of future SASSMs’ professional training and development requirements.

## Materials and methods

### Participants

SASSM School Bangladesh is a 3-day teaching initiative that has been conducted every year since 2017 except in 2020 due to COVID restrictions. However, this survey was conducted among the school participants in the years of 2017, 2018, 2019, and 2021. The program aimed at improving physicians’ knowledge and attitudes regarding sexual health and patient care. This course was specifically designed for medical practitioners from various fields, such as psychiatry, dermatology and venereology, endocrinology, urology, and obstetrics and gynecology. Before and after attending the course, a 20-item pre- (S_1_) and posttest survey (S_2_), which were prepared from lecture contents of the faculties, were rolled out to the participants.

Eligibility for the course required being a registered medical practitioner. Every new course was opened upon the board meeting of the executive committee, followed by offline and online advertisement. The course was available to attend with a minimum fee varying each year and ranging from 5000 BDT (approx. USD 42) to 10 000 BDT (approx. USD 83). This initial survey was intended to assess their baseline knowledge and attitudes. Following the training, the same participants were asked to complete an identical 20-item survey (S2) to evaluate any changes in their understanding and attitudes. The questions of the survey were taken from the resource persons of the training who gave questions for their respective topics. Participation in both surveys was voluntary and anonymous, ensuring candid and unbiased responses. Additionally, both surveys included a statement of consent for participation. No incentives were offered for completing these surveys.

### Study design

This study utilized the same participants before and after the course (pretest–posttest design). The intervention consisted of a brief, targeted training session on sexual health and related disorders. The core objectives of the training were to enhance healthcare providers’ comfort level, knowledge, and communication skills in addressing sexual health issues. The intervention included traditional didactic education and communication skill development through brief role-playing exercises. This intervention guided providers in initiating conversations about sexual health, validating patient concerns, asking contextually appropriate questions, and coordinating care. Components of the training are DSM-V classification of sexual disorders and the human sexual response cycle; sexual physiology and neuro-endocrine aspects; iatrogenic sexual disorder and sexual disorder in psychiatric practice; male and female sexual disorders and treatment; treatment of premature ejaculation (PE), erectile dysfunction (ED), and female sexual arousal and interest; disorder, genito-pelvic pain, and penetration disorder; marital and couple therapy and sex therapy; andrological emergency; and cardiac condition and sexuality and cancer and sexuality.

Both pre- and posttraining survey responses from all eligible respondents, as well as specific sub-cohorts of physicians, were analyzed who have fulfilled both pre- and posttests. The primary endpoints of the study were changes in mean scores of knowledge regarding sexual health and disorder-related issues, as measured by the surveys. Among the 203 participants of the SASSM training program, the 116 participants who completed pretest and posttest were considered as the sample size for analysis. As the sample size is 116 for assessing improvement of knowledge through training, the paired *t*-test is considered as appropriate for comparing pre- and posttest scores. In this situation, for large effect size (>0.80), the power of the test is 1.00. Logistic regression was applied to examine the influence of the training in improving knowledge. The positive difference between scores of the posttest and pretest is considered as improvement of knowledge; otherwise, no improvement of knowledge is considered as a dependent variable. All statistical tests were one-sided and conducted at a 0.05 level of significance (*P* < .05).

## Results

Among the 116 participants who completed both the pretest and posttest, most of the participants in the training were male (75%) and belonged to the age group 23 to 40 years (43%) (Table 1). The majority of the participants came from the field of psychiatry (36.7%), while the least number of participants belonged to internal medicine (2.9%).

**Table 1:** Characteristics of respondents of survey taking both pretest and posttest.

**Characteristics**	***n* (%)**
**All respondents**	116 (100.0%)
Psychiatrist	46 (39.6%)
Dermatologist	38 (32.7%)
Endocrinologist	6 (5.1%)
Urologist	14 (12.0%)
Gynecologist	10 (8.6%)
Internal medicine	2 (1.7%)
**Gender**	
Male	90 (77.5%)
Female	26 (22.4%)
**Age (in years)**	
23-40	54 (46.5%)
41-50	32 (27.5%)
51-60	25 (21.5%)
61-70	5 (4.3%)
**Experience (in years)**	
<05	59 (50.8%)
06-10	22 (18.9%)
11-15	7 (6.0%)
16-20	13 (11.2%)
>20	15 (12.9%)
**Geolocation of the participants**	
Capital (Dhaka)	90 (77.5%)
Divisional level except Dhaka	18 (15.5%)
District level	6 (5.1%)
Others	2 (1.7%)

Table 2 shows the distribution of the participants of various disciplines who attended the trainings for each year. Year-wise participants of the study were 30, 34, 28, and 24 of the training program in 2017, 2018, 2019, and 2021, respectively. The majority of the participants were from psychiatry in 2017, 2018, and 2021.

**Table 2:** Year-wise distribution of the participants who completed both surveys from various disciplines.

**Discipline**	**2017**	**2018**	**2019**	**2021**	**Total**
Psychiatrists	15	15	07	09	46
Dermatologists	14	08	09	07	38
Endocrinologists	2	00	02	02	06
Urologists	00	02	08	04	14
Gynecologists	00	08	00	02	10
Internal medicine	00	01	01	00	02
Total	30	34	27	24	116

Table 3 shows that there was a significant difference in mean score (± standard deviation) of all participants (*n* = 116) in pretest (49.6 ± 17.1) and posttest (62.3 ± 15.5) scores at the 1% level of significance. Significant improvement in knowledge was found in every year at the 1% level through the training. That is, the first alternative hypothesis (H_A1_) was accepted. The difference between the pretest and posttest means was 12.7 units, and the most knowledge gain (14.1) was in 2019 and the least knowledge gain (11.1) was in 2021.

**Table 3:** Mean score of participants according to each year.

**Year**	***n***	**Mean score**		**Knowledge gain**	***P*-value** ^**a**^
		**Pretest**	**Posttest**		
2017	30	48.1 ± 15.4	59.8 ± 15.1	11.7	<0.01
2018	34	45.6 ± 18.9	59.1 ± 15.4	13.5	<0.02
2019	28	53.7 ± 14	67.9 ± 11.9	14.1	<0.03
2021	24	52.6 ± 19.1	63.7 ± 18.7	11.1	<0.04
Overall	116	49.6 ± 17.1	62.3 ± 15.5	12.7	<0.05

a Paired *t*-test.

Table 4 shows the impact of the socio-demographic factors (gender, location, age (in years), and experience (in years)) on improvement of knowledge among the participants after the training session. It is observed that none of the socio-demographic has a significant impact on the improvement of knowledge as *P*-values of the odds ratio and adjusted odds ratio are > .05. That is, the second alternative hypothesis (H_A2_) was rejected.

**Table 4:** Impact of socio-demographic variables on improvement of knowledge through training.

**Variables**	**Odds ratio**	**95% C.I.**		***P*-value** ^**a**^	**Adjusted odds ratio**	**95% C.I.**		***P*-value** ^**a**^
		**Lower**	**Upper**			**Lower**	**Upper**	
Gender (Ref female)	0.68	0.076	6.093	0.73	0.497	0.051	4.816	0.546
Location (Ref Mega city)	1.79	0.201	15.972	0.602	1.017	0.095	10.841	0.989
Age (in years)	0.919	0.843	1.001	0.053	0.916	0.721	1.163	0.471
Experience (in years)	0.913	0.829	1.005	0.062	1.000	0.747	1.339	0.999

a Logistic regression (dependent variable: improvement of knowledge through training (yes or no) is yes when score of posttraining is higher than that of pretraining otherwise no.

## Discussion

The main aim of this study was to measure the impact it made on the participants in terms of knowledge in a cost-effective setup for an under-resourced country where the practice of sexual medicine is only comparable to sailing a boat by a novice boatman without a compass. Scientific interest in the subject of sexual medicine has grown over the past 20 years as a result of improvements in our knowledge of human sexuality, and the function and malfunction of the sexual organs.^9^ However, there were two major barriers standing in the way of effectively communicating these developments to the wider public. First, the methods for spreading new ideas in the community were undefined to scientists working in the field of sexual medicine^10^ and second, the general public lacked the readiness to confront the current “conspiracy of silence” in sexual field.^11^ Furthermore, it appeared that research results were not being properly translated into clinical practice as a result of a lack of organized sexual medicine education and training options for health professionals.^12^ The goal of the training session is to build a connection between the scientific and academic disciplines of sexual medicine and the community.

The 57% response rate of survey completion in the present study is comparable to other studies’ response rates, which varied between 20% in Italy and 80% in Sweden.^13^ Compared to the existing sexologist surveys, SASSM has a substantially higher percentage of men, nearly 75%. It surpasses the percentage of physicians who identify as sexologists or sexual therapists, which spanned from 25% in Italy to 70% in Sweden.^14^ The feminization of medicine has grown more prominent in Europe but less so globally.^15^ In the stereotypically male field of sexual medicine, that might be especially true. Despite previous research indicating that the majority of physicians practicing sexual medicine in France and Denmark are general practitioners, gynecologists in the United Kingdom, and urologists in Finland and Italy,^16^ this group has an exceptionally high proportion of psychiatrists. It is also apparent that this inclination reflects the significant number of psychiatric experts who attended the course, given that it was planned and led by a number of distinguished psychiatry faculty members.

As sexual dysfunction is extremely common, it is critical to educate medical professionals to properly deal with the health burden caused by sexual dysfunction in order to enhance sexual health outcomes and develop more doctors as leaders in sexual medicine.^17^ Medical education often fails to adequately prepare future physicians for their roles as sexual health caregivers, leaving them ill-equipped to address the challenges and complexities of sexuality during their undergraduate education.^18^ Furthermore, despite the fact that medical students are regularly taught how to take sexual histories, this knowledge is usually forgotten throughout clinical rotations and training when time restrictions or other physicians discourage taking sexual histories.^19^ Consequently, training on sexual health should continue during and after postgraduate training.

### Strengths

Participants from multiple disciplines were involved in the training session, which enhanced its relevance and applicability across various healthcare fields, particularly in psychiatry and dermatology. The training session demonstrated improvement in knowledge across the years, which reflects the reliability and effectiveness of the training program.

Strong statistical evidence from the pre- and posttest analysis also validates the impact of the training sessions.

### Limitations

The gender and age imbalance, with 75% male participants and the majority aged between 23 and 40 years, limits the findings to use as generalized for all. Less participation from a few fields, such as internal medicine (2.9%), reduces the applicability of the results to other disciplines.

The lack of assessment of long-term knowledge retention or the practical application of training outcomes further constrains the generalizability of the results.

## Conclusion

The results of this study indicate that knowledge of sexual medicine among participants was increased by more than 12.7 units, and the gain of knowledge was more significant at the end of the SASSM program than it was at the beginning. Thus, the first alternative hypothesis was accepted. It is also found that there was no impact of socio-demographic variables (gender, location, age, and experience) on knowledge gain. Thus, the second alternative hypothesis was rejected. It is also indicating that the training program and its content were the main cause of improvement of knowledge. Physicians of sexual medicine have a unique opportunity to learn and demonstrate their knowledge through the SASSM course, the first of its type in Bangladesh. As the result indicates the outcome was persistently significant over years, it implies the notion that the course can be replicated in similar socio-economic contexts with some cultural adaptation anywhere in the world. SASSM as an organization operates under the belief that by setting higher standards for training, it will likewise set higher standards for care. This study verifies that the initial steps have been taken down that path, but much more has to be done.

## References

[1] Chew PY, Choy CL, Sidi HB, et al. The association between female sexual dysfunction and sexual dysfunction in the male partner: a systematic review and meta-analysis. J Sex Med. 2021;18(1):99–112. 10.1016/j.jsxm.2020.10.001 33303390

[2] Irfan M, Hussain NHN, Noor NM, Mohamed M, Sidi H, Ismail SB. Epidemiology of male sexual dysfunction in Asian and European regions: a systematic review. Am J Mens Health. 2020;14(4):1557988320937200. 32623948 10.1177/1557988320937200 PMC7338652

[3] Abdelatti SI, Ismail RM, Hamed RA. ‘Sexual dysfunctions in a sample of male psychiatric patients compared to medically ill patients’. Middle East current. Psychiatry. 2020;27:1–10.

[4] Nganzo MS . Perceptions of Sexually Transmitted Infections in Select Youth and Young Adult African Newcomers in Regina, Saskatchewan Master's thesis. Saskatoon (Canada): University of Saskatchewan; 2021.

[5] Ahsan MS, Arafat SY, Ali R, Rahman SA, Ahmed S, Rahman MM. Sexual history taking competency: a survey among the clinicians in Bangladesh. International Journal of Psychiatry. 2016;1(1):4. 10.33140/IJP/01/01/00006

[6] Monaghan L, Gabe J. editors Key Concepts in Medical Sociology. 3rd ed. London: Sage Publications Ltd; 2022.

[7] Reisman Y et al. ‘New developments in education and training in sexual medicine’, the journal ofSexual medicine. Elsevier Masson SAS. 2013;10(4):918–923. 10.1111/jsm.12140 23551541

[8] Lewis RW . Comprehensive history of the International Society for Sexual Medicine. Sexual Medicine Reviews. 2021;9(4):517–541. 10.1016/j.sxmr.2021.03.004 34373235

[9] Goldfarb ES, Lieberman LD. Three decades of research: the case for comprehensive sex education. J Adolesc Health. 2021;68(1):13–27. 10.1016/j.jadohealth.2020.07.036 33059958

[10] Farsi D . Social media and health care, part I: literature review of social media use by health care providers. J Med Internet Res. 2021;23(4):e23205. 10.2196/23205 33664014 PMC8056296

[11] Goldblatt H, Band-Winterstein T, Lev S, Harel D. “Who would sexually assault an 80-year-old woman?” barriers to exploring and exposing sexual assault against women in late life. Journal of interpersonal violence. 2022;37(5-6):2751–2775. 10.1177/0886260520934440 32627631

[12] Kirana P et al. ‘A conceptual framework for the evolution of sexual medicine and a model for the development of alternative sexual health services: 10-year experience of the Center for Sexual and Reproductive Health’, the journal of sexual medicine. Elsevier Masson SAS. 2009;6(9):2405–2416. 10.1111/j.1743-6109.2009.01320.x 19453879

[13] Simonelli C et al. Sexology as a profession in Europe : results from an Italian survey La sexologie comme profession en Europe : résultats d. une enquête italienne. 2006;15(1):50–7. 10.1016/j.sexol.2005.11.008

[14] Fugl-meyer KS, Giami A. Swedish clinical sexologists. Who are they? Who do they treat? Les Sexologues cliniciens suédois qui sont-ils o. 2006;15(1):14–21. 10.1016/j.sexol.2005.11.003

[15] Phillips SP, Austin EB. The feminization of medicine and population health. Jama. 2015;301(8):863–864. 10.1001/jama.2009.155 19244195

[16] Michaud PA, Jansen D, Schrier L, Vervoort J, Visser A, Dembiński Ł. An exploratory survey on the state of training in adolescent medicine and health in 36 European countries. Eur J Pediatr. 2019;178:1559–1565. 10.1007/s00431-019-03445-1 31463767 PMC6733827

[17] Giritharan S . Socio-Cultural Perspectives, Challenges, and Approaches to Sexual Health in the Indian Subcontinent Rowland DL and Jannini EA, eds. Cultural Differences and the Practice of Sexual Medicine: A Guide for Sexual Health Practitioners; 2020: 39–61.

[18] Van Der Vlugt I . ‘Comprehensive sexuality education knowledge file index’. Rutgers International, December. 2018: 1–31. 10.3390/v10110641

[19] Beebe S, Payne N, Posid T, et al. The lack of sexual health education in medical training leaves students and residents feeling unprepared. J Sex Med. 2021;18(12):1998–2004. 10.1016/j.jsxm.2021.09.011 34711518

